# Atomic-scale interactions between quorum sensing autoinducer molecules and the mucoid *P. aeruginosa* exopolysaccharide matrix

**DOI:** 10.1038/s41598-022-11499-9

**Published:** 2022-05-11

**Authors:** Oliver J. Hills, Chin W. Yong, Andrew J. Scott, Deirdre A. Devine, James Smith, Helen F. Chappell

**Affiliations:** 1 School of Food Science & Nutrition, University of Leeds, Woodhouse Lane, Leeds, LS2 9JT UK; 2 Daresbury Laboratory, Scientific Computing Department, Science and Technology Facilities Council, Keckwick Lane, Daresbury, Warrington WA4 4AD UK; 3 Division of Pharmacy and Optometry, School of Health Sciences, University of Manchester, Oxford Road, Manchester, M13 9PL UK; 4 School of Chemical & Process Engineering, University of Leeds, Woodhouse Lane, Leeds, LS2 9JT UK; 5 School of Dentistry, University of Leeds, Clarendon Way, Leeds, LS2 9LU UK

**Keywords:** Density functional theory, Molecular dynamics, Structure prediction, Computational biophysics, Biopolymers in vivo, Biofilms

## Abstract

Mucoid *Pseudomonas aeruginosa* is a prevalent cystic fibrosis (CF) lung coloniser whose chronicity is associated with the formation of cation cross-linked exopolysaccharide (EPS) matrices, which form a biofilm that acts as a diffusion barrier, sequestering cationic and neutral antimicrobials, and making it extremely resistant to pharmacological challenge. Biofilm chronicity and virulence of the colony is regulated by quorum sensing autoinducers (QSAIs), small signalling metabolites that pass between bacteria, through the biofilm matrix, regulating genetic responses on a population-wide scale. The nature of how these molecules interact with the EPS is poorly understood, despite the fact that they must pass through EPS matrix to reach neighbouring bacteria. Interactions at the atomic-scale between two QSAI molecules, C_4_-HSL and PQS—both utilised by mucoid *P. aeruginosa* in the CF lung—and the EPS, have been studied for the first time using a combined molecular dynamics (MD) and density functional theory (DFT) approach. A large-scale, calcium cross-linked, multi-chain EPS molecular model was developed and MD used to sample modes of interaction between QSAI molecules and the EPS that occur at physiological equilibrium. The thermodynamic stability of the QSAI-EPS adducts were calculated using DFT. These simulations provide a thermodynamic rationale for the apparent free movement of C_4_-HSL, highlight key molecular functionality responsible for EPS binding and, based on its significantly reduced mobility, suggest PQS as a viable target for quorum quenching.

## Introduction

*Pseudomonas aeruginosa* is a Gram-negative bacterium capable of colonising a wide variety of different environments and habitats. The cystic fibrosis (CF) lung is one such environment and colonisation by *P. aeruginos*a is highly prevalent, leading to increased mortality in CF patients^1^. *P. aeruginosa* lung infections currently account for the majority of the morbidity and mortality seen in CF patients^2^ and the chronicity of *P. aeruginosa* infections is associated with the bacterium’s ability to form a biofilm^3^. Bacterial biofilms are comprised of colonies of bacterial cells enveloped within an extracellular matrix (ECM)^4^. The ECM itself encompasses polysaccharide, protein, lipid and nucleic acid constituents, referred to collectively as the *matrixome*^5^. Explanted lungs of deceased CF patients reveal that it is the mucoid *P. aeruginosa* phenotype that is primarily responsible for destruction of the CF lung^6^. The mucoid phenotype is characterised as exopolysaccharide (EPS) alginate overproducing, where its matrixome is primarily composed of linear, acetylated, anionic alginate^7–10^, cross-linked by calcium (Ca^2+^), an ion significantly elevated in the CF lung^11^. Recently, we have used quantum chemical Density-Functional Theory (DFT) to construct molecular models, structurally representative of the mucoid *P. aeruginosa* EPS, that prove Ca^2+^ can induce highly stable EPS aggregation relative to other biological ions elevated in CF sputum^12^.

The formation of stable *P. aeruginosa* cation cross-linked biofilm matrices relies on the quorum sensing signalling pathway. Quorum sensing (QS) is a mechanism where individual bacterial cells perceive the cell density in their local environment and, in turn, coordinate gene expression on a population-wide scale^13^. It is a mechanism reliant upon the production of cell-to-cell signals, called quorum sensing autoinducer (QSAI) molecules, leading to biofilm matrix proliferation^14^. When a single bacterium releases a QSAI molecule, it is at too small a concentration to be detected by neighbouring bacterial cells. However, if QSAI molecules are collectively released by enough bacteria, the concentration of these molecules increases past a threshold level, allowing the bacteria to recognise a critical cell mass and activate specific genes^15^. Specifically, for *P. aeruginosa* aggregates, QSAI release from approximately 2000 cells is required to initiate QS^16^. QS, effectively, is the mechanism underlying bacterial cell-to-cell communication and, importantly, is a mechanism active in CF lungs infected with *P. aeruginosa*^17^. Confocal laser scanning microscopy has identified the existence of three distinct ECM layers surrounding naturally occurring bacterial sub-populations embedded within biofilm matrices^18^. The first is a layer surrounding individual bacterial cells, the second a layer separating individual cells (intercellular ECM) and the third a layer separating different sub-populations^18^. Considered along-side observations that QSAI molecules can partition into the biofilm matrix^19^, the implication is that the QSAI molecules must pass through ECM material to reach neighbouring bacteria. The type and variety of molecular interactions that facilitate the movement of QSAI’s through the ECM is not understood and these molecules are simply assumed to be freely diffusible^20,21^.

The vast majority of Gram-negative bacteria utilise acetylated homoserine lactones (HSL) as their primary QSAI molecules^15^, which are typically specific to the LasR and/or RhlR transcriptional activators^22^. Specifically, C_4_-HSL and 3-oxo-C_12_-HSL are the two primary HSL based QSAI molecules utilised by *P. aeruginosa* in the CF lung^17,23^. The transcriptional regulators RhlR and LasR are bound by C_4_-HSL and 3-oxo-C_12_-HSL respectively in order to regulate the expression of several genes^24,25^, and transcriptome analysis has determined that between 6 and 10% of the *P. aeruginosa* genome is regulated by these systems^26^. C_4_-HSL is a QSAI molecule that, most notably, plays a significant role in biofilm (EPS) proliferation, maturation and virulence factor expression in *P. aeruginosa* biofilms^24,27^. There also exists a third key QSAI molecule, the *Pseudomonas* quinolone signal (PQS)^28^, which is also present in the lungs of infected CF patients^29^. This signal is found in higher concentrations when bacterial cultures reach the late stationary phase of growth^30^ and therefore plays a major role in biofilm maintenance. For example, PQS production is able to regulate the autolysis of cells^31^, assist in iron (Fe^3+^) sequestration^32–34^ and induce rhamnolipid production^35^.

**Figure 1:**
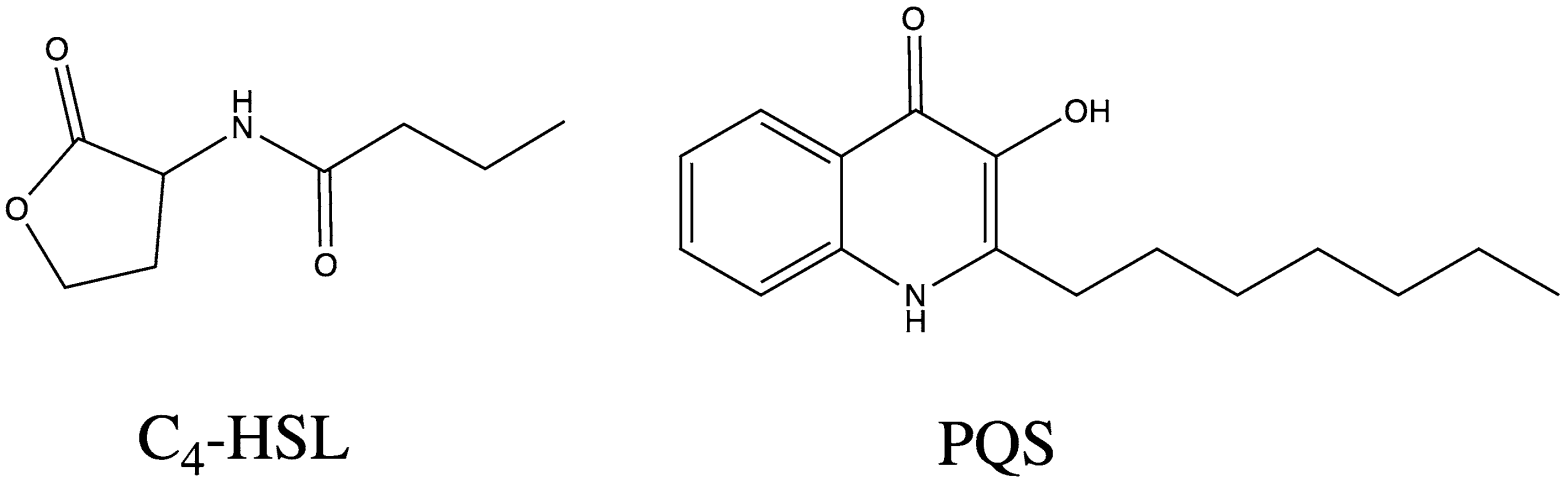
Molecular structures of C_4_-HSL and PQS.

3-oxo-C_12_-HSL is a QSAI molecule most implicated in the initial differentiation to the biofilm mode of life upon deposition within the CF lung, with this molecule’s role becoming negligible once bacteria have established a firm attachment to the substratum^14,24,27^. Conversely, C_4_-HSL and PQS (Fig. 1) are two QSAI molecules important for establishing mature biofilms and therefore contributing to biofilm chronicity. They are molecules that will move throughout the EPS and are ideal candidates to study possible modes of interaction with the EPS matrix. The intent of this work is to rationalise which molecular interactions dictate the motion of these molecules through the EPS, beyond the explanation of simple diffusion. The use of in silico theoretical modelling, specifically molecular dynamics and molecular docking, has been used primarily to study the origin of interactions between QS inhibitor molecules and the QS regulatory proteins LasR and RhlR^36–38^. However, there has not been application of any theoretical techniques to study the nature and origin of the interactions, at the atomic-scale, between QSAI molecules and EPS, despite its obvious connection to pharmaceutical design and EPS penetration. Therefore, the purpose of this investigation is to identify the key molecular functionality of QSAI molecules, secreted by mucoid *P. aeruginosa* in the CF lung, which mediate interactions with the EPS matrix. To this end, finite temperature explicit solvent molecular dynamics (MD) has been employed to identify QSAI-EPS adducts which are representative of those that occur at physiological equilibrium. Combined with DFT, to ensure accurate energy calculations of QSAI-EPS systems, post-simulation thermodynamic stabilities and binding energies of such adducts have been accurately evaluated and molecular functionality pertinent for binding elucidated at the atomic-scale.

## Results

### Exothermic association of four EPS chains about Ca^2+^ ions

As detailed in the “Materials and methods” section, a DFT procedure was employed to develop an exothermic calcium cross-linked 4-chain EPS network suitable for studying EPS-QSAI interactions. This 4-chain EPS model (either 4-PolyMG or 4-PolyM) was constructed through the fibrillar stacking of smaller Ca^2+^ cross-linked 2-chain EPS units (either two 2-PolyMG or two 2-PolyM systems).

The formation energies (evaluated according to Eq. (1)) for each stacking arrangement in the 4-PolyMG and 4-PolyM systems are given in Tables S1 and S2 respectively**.** In both 4-chain systems, all stacking arrangements are thermodynamically stable (all possesses negative formation energies), which shows that, independent of in which orientation the 2-chain systems aggregate, having two 2-chain systems in the vicinity of one another, and/or combining to form a larger 4-chain system, is thermodynamically stable.

The number of ionic bonds established between the adjoining stacks (Tables S1, S2) correlates well with overall thermodynamic stability, with the 4-chain systems that establish the largest and smallest number of ionic bonds corresponding to the most and least thermodynamically stable systems respectively. Furthermore, stacking arrangements that establish an equal number of ionic bonds between adjoining stacks are comparatively as stable, independent of the number of hydrogen bonds also established. This, in turn, highlights that the formation of exothermic 4-chain complexes is driven more through the formation of ionic interactions rather than hydrogen bonding interactions. Previous DFT modelling has highlighted that exothermic association of multiple algal alginate disaccharides about divalent cations is driven through ionic interactions also^39^ and this work extends this observation to bacterial alginates (the mucoid *P. aeruginosa* EPS).

The thermodynamic stability of the 4-PolyMG system increases as the number of acetyl groups facing the adjoining stack decreases and the number of ionic bonds between adjoining stacks subsequently increases. Having both acetyl groups facing away from the adjoining stack minimises steric repulsion and facilitates the close association of four chains about Ca^2+^ ions. Interestingly, it is thermodynamically stable to have two 2-PolyMG chains in the same vicinity without interacting, for example, when both acetyl groups face towards the adjoining stack. Similar observations have been made in MD simulated annealing studies investigating Ca^2+^ induced association of three copolymeric $\beta$-d-mannuronate-$\alpha$-l-guluronate decamers, which also identify multi-chain aggregations whereby the third chain lies in the vicinity of a Ca^2+^ cross-linked 2-chain system without directly binding^40^. In our models, this is a feature, driven by acetyl steric repulsion, that creates void spaces within the EPS. Structurally, *P. aeruginosa* biofilms can be described as open systems encompassing cells, extracellular matrix material and void spaces^41^, where the latter act as channels allowing the flow of water throughout the biofilm^42^. All stacking arrangements lead to ionic association of four chains in the 4-PolyM system, therefore, the presence of acetylated mannuronate–guluronate blocks in the EPS provides a structural origin for void spaces and water channels in mucoid *P. aeruginosa* biofilms.

**Figure 2:**
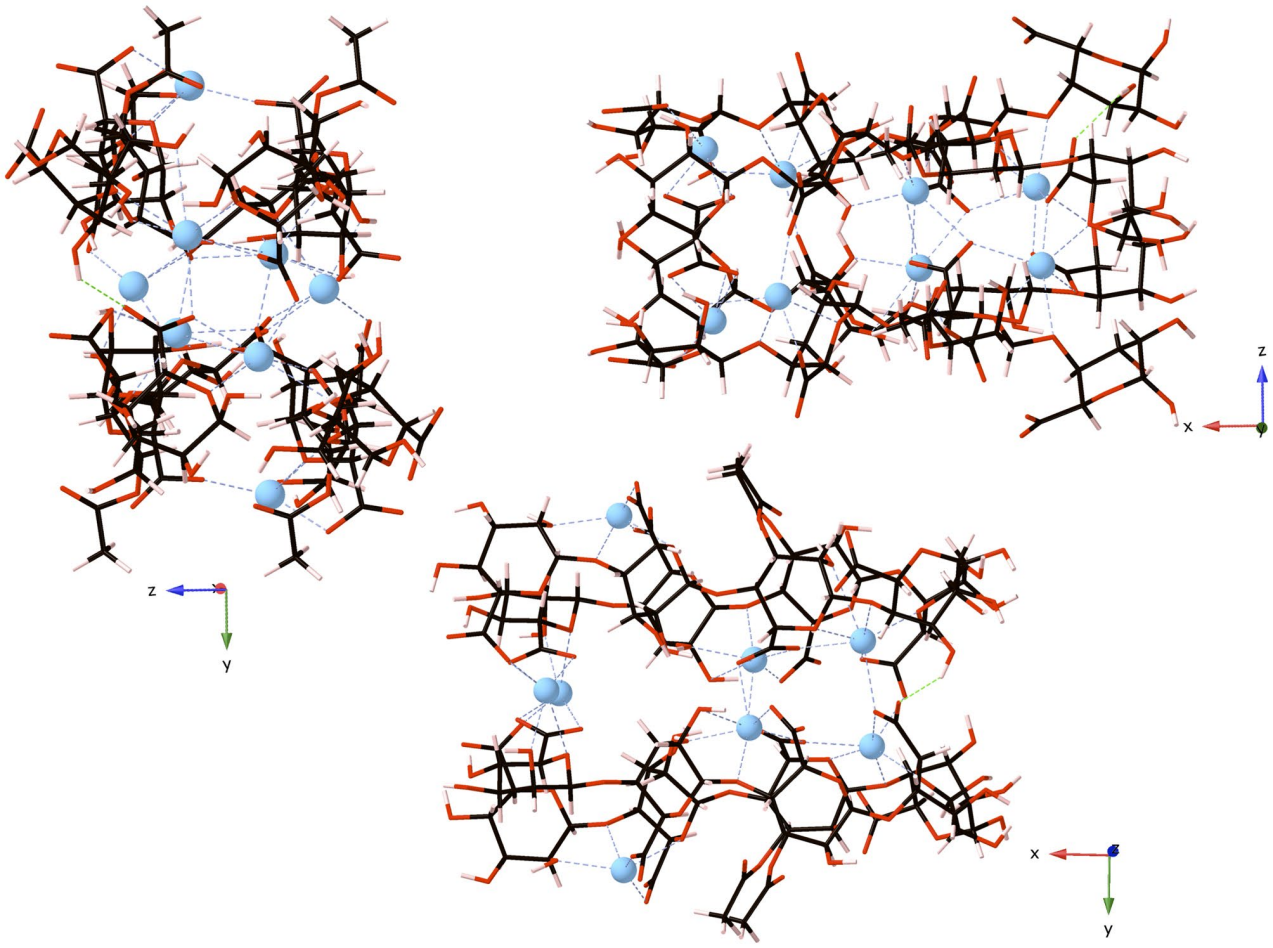
The 4-PolyMG* structure, corresponding to the most thermodynamically stable arrangement of four EPS chains complexed about Ca^2+^ ions, viewed down the x, y and z axes. Carbon atoms are shown in grey, oxygen in red, calcium in blue and hydrogen in pink. Calcium–oxygen ionic bonds are shown with dashed blue lines and hydrogen bonds are shown with dashed green lines.

Following subsequent DFT simulations of the 4-PolyM and 4-PolyMG systems, it is clear that the 4-PolyMG* system (Fig. 2) is slightly more thermodynamically stable relative to the 4-PolyM* system (Fig. S4). The stability difference is minor because these 4-chain complexes share highly similar geometrical features. Specifically, the average O-Ca^2+^, COO-Ca^2+^, OH-Ca^2+^, Glycosidic O-Ca^2+^ and Ring O-Ca^2+^ bond lengths in the 4-PolyMG* system are highly similar to those observed in the 4-PolyM* system (Table S3). The only ionic contact present in the 4-PolyM* system that isn’t present in the 4-PolyMG* system is the acetyl O-Ca^2+^ contact. Ionic contacts to acetyl groups, therefore, are a geometric feature that distinguishes multi-chain, exothermic, ionic association of acetylated mannuronate EPS fractions from acetylated mannuronate–guluronate EPS fractions. Acetyl groups are involved in ionic association in the former, but are implicated in increased steric repulsion and preventing ionic association in the latter. The slight difference in stability between these two systems is rationalised when considering the additional ionic contact established between adjoining stacks, the number of calcium ions involved in the formation of ionic bonds between adjoining stacks and their coordination numbers (CN). Higher maximum coordination numbers are observed in the 4-PolyMG* system (CN = 7), which also encompasses an additional ionic bond between the adjoining stacks, compared to the 4-PolyM* system (CN = 6). Furthermore, five Ca^2+^ ions are involved in establishing ionic bonds between the adjoining stacks in the 4-PolyMG* system, whereas only four are involved in the 4-PolyM* system. Overall, these three factors outline a larger 4-chain intracomplex space in the 4-PolyMG* system, compared to the 4-PolyM* system, making it more stable.

Within the 4-PolyMG* system, the COO-Ca^2+^ contacts are the ionic bonds that are most stable (shortest of all O-Ca^2+^ bonds with the largest bond populations; Table S3) and occur most frequently (Fig. 2). Therefore, the COO group is the functional group most implicated in the formation of an exothermic 4-chain complex, correlating well with previous findings in MD simulations of Ca^2+^ induced algal alginate gelation^43–45^. Ca^2+^ ions with CN = 7 are coordinated to four different uronate residues, whereas when the CN = 5 or 6, the Ca^2+^ ions are only bound to three uronate residues. Coordination to four uronate residues per Ca^2+^ ion is closer to the classical *egg-box* description of calcium chelation by alginates^46^. Previous DFT modelling in the group has highlighted that calcium chelation by two bacterial alginate chains deviates from the egg-box model^12^, so it is interesting to observe egg-box characteristics regained as the number of coordinating bacterial alginate chains increases. Even though coordination to four uronate residues is possible, no Ca^2+^ ion is bound to more than three EPS chains. The implication of this, is that, 3-chain complexation is possible about a single Ca^2+^ ion, but 4-chain complexation is not; $\ge$ 2 Ca^2+^ ions are needed to establish 4-chain aggregation. Finally, returning to the discussion on void spaces, fibrillar stacking of two 4-PolyMG* systems, to form an 8-chain system, would recover a stacking arrangement where acetyl groups face towards the adjoining stack—creating a void space. Therefore, this further highlights, in acetylated mannuronate–guluronate EPS fractions, the formation of void spaces is inevitable.

### Physiological EPS structure

**Figure 3:**
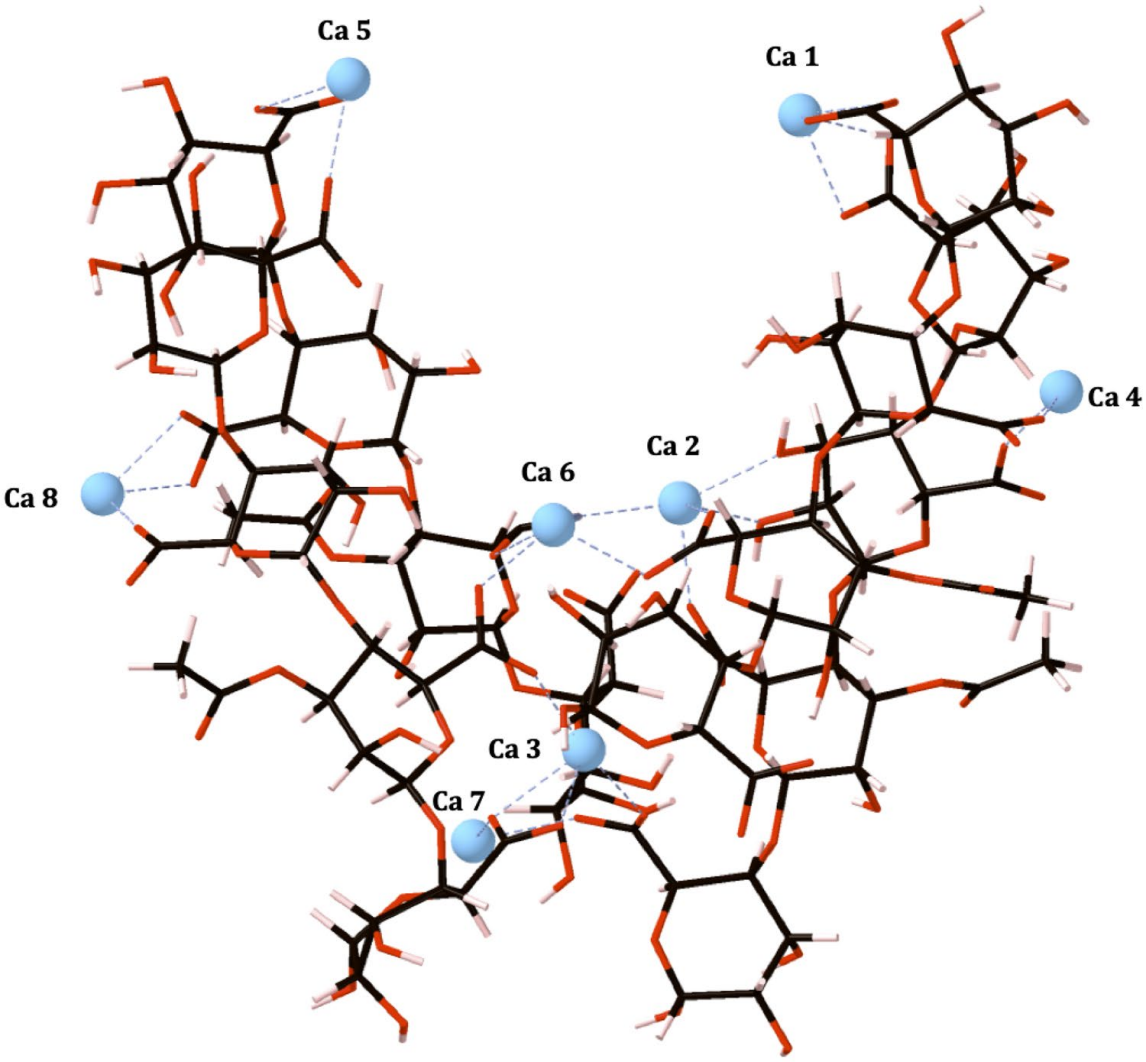
The 4-PolyMG_MD_ structure, corresponding to the structure of the hydrated mucoid *P. aeruginosa* EPS at physiological temperature. Carbon atoms are shown in grey, oxygen in red, calcium in blue and hydrogen in pink. Calcium ions are labelled and calcium–oxygen ionic bonds are shown with dashed blue lines. Explicit water molecules are not shown.

The 4-PolyMG_MD_ structure, which corresponds to the structure of the hydrated mucoid *P. aeruginosa* EPS at physiological temperature, is shown in Fig. 3.

Immediately, it is clear that there is a significant deviation away from the neatly stacked EPS chains obtained from the DFT modelling and the adoption of an entangled V-shaped motif. This motif isn’t unexpected, as it has also been observed in MD simulations of calcium algal alginates^40^. The creation of a V-shaped cleft, introduces a structural discontinuity into the EPS, which assists in rationalising the complex, discontinuous, EPS structures observed in transmission electron microscopy (TEM) measurements on *P. aeruginosa* biofilms^47^. Interestingly, the V-shaped cleft, perhaps, also provides an origin for the large-scale branched/dendritic organisation of *P. aeruginosa* EPS scaffolds^48^.

The increased conformational flexibility allows the system to undergo torsional change which maximises the establishment COO-Ca^2+^ interactions. The frequency of COO-Ca^2+^ interactions (25 contacts) far exceed the frequency of any other O-Ca^2+^ contact, with the only other Ca^2+^ coordinating oxygen functional group being the hydroxyl group (OH-Ca^2+^; 2 contacts). The OH-Ca^2+^, glycosidic O-Ca^2+^ and ring O-Ca^2+^ interactions, the latter two absent from the 4-PolyMG_MD_ structure, are comparatively as stable in the 4-PolyMG* structure (Table S3) and are eliminated from the 4-PolyMG* structure upon thermal equilibration at 310 K as a result of the EPS chains favouring the establishment of COO-Ca^2+^ above all other oxygen-Ca^2+^ interactions. Carboxylate groups being the dominant contributor to the Ca^2+^ chelation geometry has been observed extensively in finite temperature MD simulations of solvated calcium alginate networks^40,43–45,49^. Combined with the 4-PolyMG_MD_ structure obtained in this work, it is clear how the presence of temperature and solvent reduces the diversity in the geometry of the chelation site within Ca^2+^ chelate pockets and highlights that the preferred mode of aggregation under physiological conditions is through the carboxylate groups.

The only structural feature retained from the 4-PolyMG* system, is the acetyl groups facing away from the neighbouring stack. Therefore, there has not been a complete (180°) inversion about the chain axes in any of the EPS chains. The acetyl groups prefer to face the solvent environment than face the neighbouring stack which, in turn, reinforces the tendency of the acetyl groups to orient further away from the main EPS chains. Only Ca 2 and Ca 6 (Fig. 3) are bound to three EPS chains—all other calcium ions are involved in coordination to two EPS chains. More calcium ions were bound to three EPS chains in the exothermic 4-PolyMG* structure and, therefore, in the 4-PolyMG_MD_ structure it is clear that the intracomplex space is heavily reduced upon thermal equilibration at physiological temperature. Effectively, the exothermic 4-chain system (4-PolyMG*) has partitioned into two sets of 2-chain systems (2 $\times$ 2-PolyMG systems) with only Ca 2 and Ca 6 keeping the bacterial alginate network together. The reduction in the size of the intracomplex space is the reason for the origin of V-shaped cleft. The structural morphology of the 4-PolyMG_MD_ system, correlates well with morphologies of calcium cross-linked mannuronate–guluronate heteropolymers observed in recent large-scale multi-chain implicit solvent MD simulations^50^. In these simulations, heteropolymers (copolymeric mannuronate–guluronate) have increased chain flexibility relative to homopolymers (polymannuronate) which leads to entanglement upon calcium cross-linking and giving discontinuous, V-shaped, morphologies possessing open clefts after aggregation^50^.

### QSAI simulations

**Figure 4:**
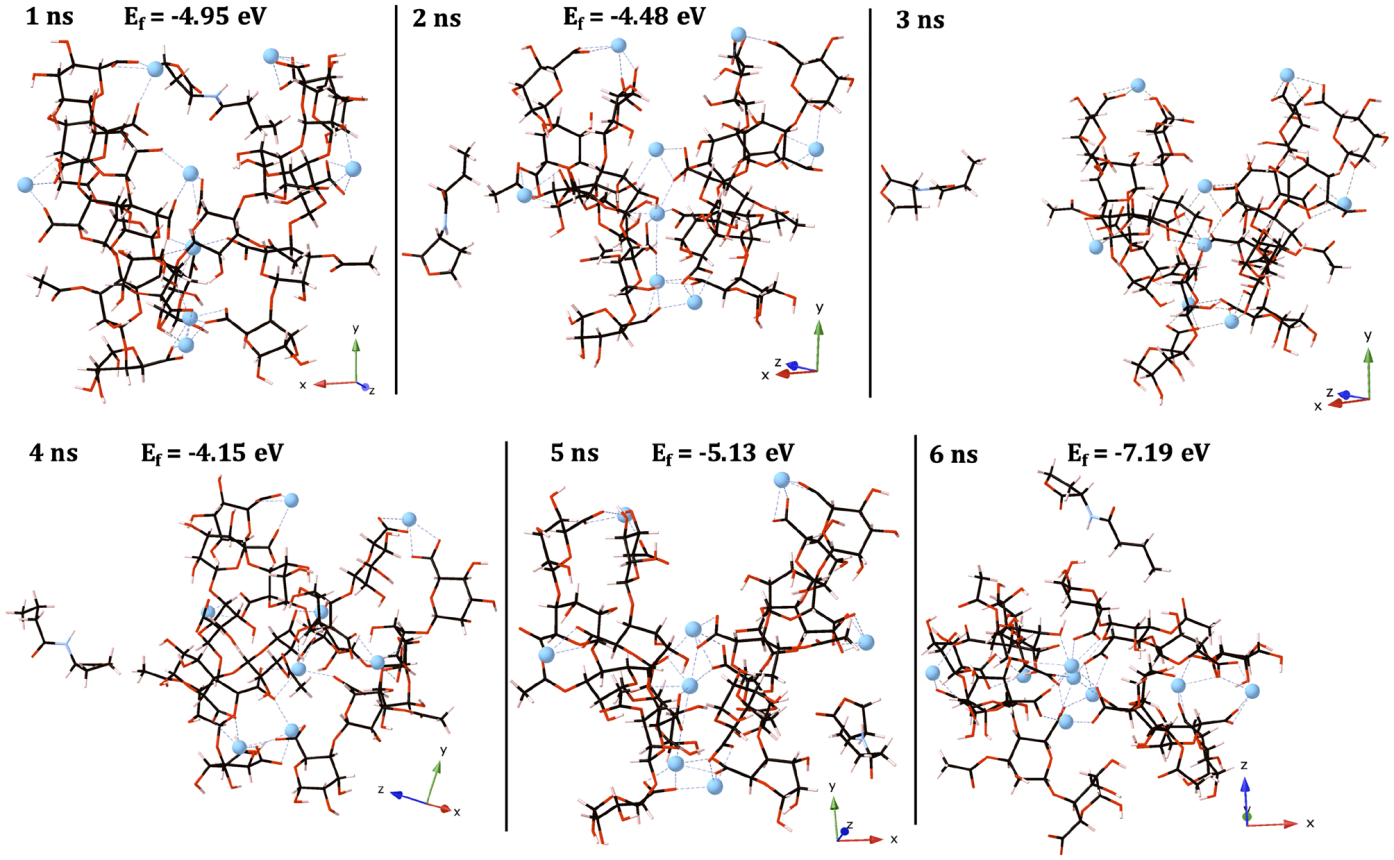
EPS-C_4_-HSL structures and their formation energies (eV). Carbon atoms are shown in grey, oxygen in red, calcium in blue and hydrogen in pink. Calcium–oxygen ionic bonds are shown with dashed blue lines. Formation energies weren’t calculated if the molecule > 6 Å away from the EPS.

**Figure 5:**
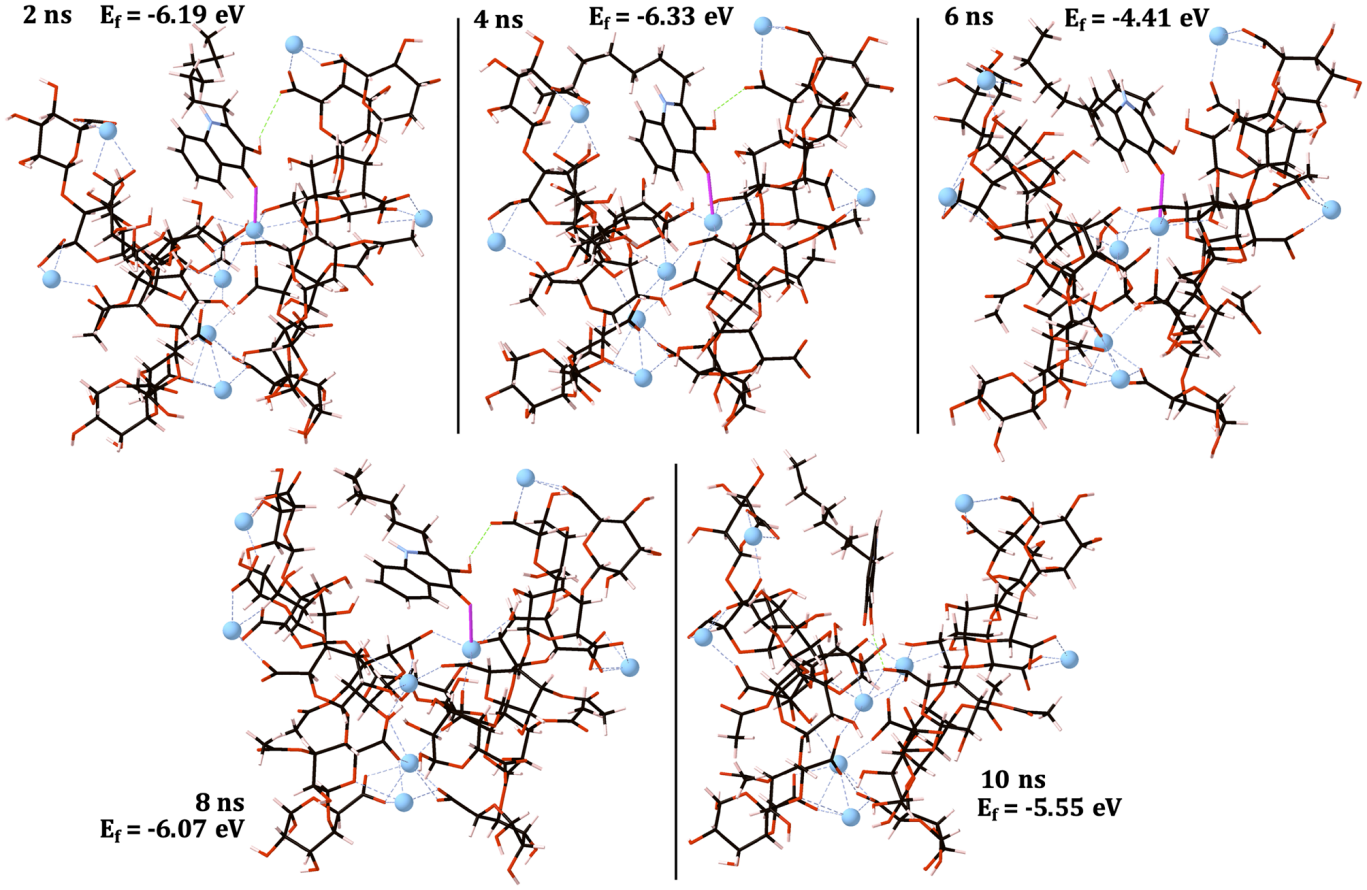
EPS-PQS structures and their formation energies (eV) displayed at 2 ns intervals. Carbon atoms are shown in grey, oxygen in red, calcium in blue and hydrogen in pink. Calcium–oxygen ionic bonds are shown with dashed blue lines and hydrogen bonds are shown with dashed green lines. Ionic bonds between the PQS and 4-PolyMG_MD_ system are shown with bold pink lines.

EPS-C_4_-HSL and EPS-PQS structures and their formation energies are shown in Figs. 4 and 5 respectively. The PQS molecule binds early and remains bound to the EPS throughout the full time-scale of the trajectory and, therefore, EPS-PQS adducts are displayed at 2 ns intervals for brevity (Fig. 5).

The C_4_-HSL molecule fails to show any molecular interactions with the EPS. The C_4_-HSL molecule liberates the cleft and migrates over the EPS interface, but after 6 ns (Fig. 4) becomes separated from the EPS and does not return. In contrast, the PQS molecule forms both an ionic interaction between its ketone oxygen and a single Ca^2+^ ion and a hydrogen bonding interaction between its hydroxyl group and a single EPS carboxylate group (Fig. 5). Consequently, the PQS molecule forms a thermodynamically stable ionic complex with the EPS. In fact, these interactions are sufficient to keep the PQS molecule *tethered* in the cleft of the 4-PolyMG_MD_ structure, overcoming hydrophobic repulsion between the hydrocarbon and EPS chains, for the full time-scale of the trajectory. After 8 ns, the ionic ketone O-Ca^2+^ interaction is lost and is compensated for by a rearrangement in the single hydrogen bonding interaction, which keeps the PQS molecule bound within the cleft. The EPS-PQS adduct at 6 ns, where the hydroxyl hydrogen bond is absent and only the ketone O-Ca^2+^ remains, is the least thermodynamically stable configuration and, interestingly, is less stable than the EPS-PQS adduct at 10 ns where only the hydroxyl hydrogen bond exists. Fluorescence resonance energy transfer (FRET) investigations of PQS incorporation into lipopolysaccharide (LPS) scaffolds have shown the hydroxyl group to be critically important for PQS–LPS interactions^51^ and these simulations further call attention to the role of the PQS hydroxyl group in molecular binding. The propensity of the PQS molecule to form ionic and hydrogen bonding interactions with the EPS significantly reduces its mobility and, conversely, the C_4_-HSL molecule, which forms no such interactions, is notably more mobile.

Despite the C_4_-HSL molecule sharing similar functional groups to PQS, namely, two ketone functional groups, one located on its “head” group and the other as part of an amide linkage attaching the hydrocarbon tail, neither of these groups form any ionic interactions with the EPS Ca^2+^ ions. The relative hydrophobicity of C_4_-HSL is smaller than PQS, the latter of which possesses conjugated aromatic rings and a larger hydrocarbon tail. It is, therefore, no surprise that the C_4_-HSL molecule partitions more readily into bulk solvent. Biofilms are heavily hydrated systems, with the major matrix component being water^52^. The C_4_-HSL molecule has the disposition to readily move into the solvent phase and consequently into void spaces filled with water, in preference to remaining in the EPS vicinity. Now, in this circumstance, its transport is assisted by convection^53^ and its diffusibility increases as a result of decreased tortuosity^54^. Along with the absence of any molecular interactions with the EPS, this offers *non-local* behaviour and gives the molecule the ability to travel further distances throughout the biofilm. By contrast, the PQS molecule shows a preference for remaining in the EPS vicinity for the full time-scale of its trajectory. The PQS ketone and hydroxyl groups are heavily implicated in molecular interactions with the EPS and our simulations would suggest that PQS is, potentially, a short range QSAI signal. In fact, this complements recent secondary ion mass spectroscopy and confocal Raman microscopy studies into spatial distributions of alkyl quinolones in *P. aeruginosa* biofilms, which also expose PQS as a local QSAI signal^55,56^. AQNO, an alkyl quinolone structural analogue of PQS, where the ketone and hydroxyl groups are absent, had the larger spatial distribution^56^, thus, offering a link, reinforced by our simulations, between the PQS ketone and hydroxyl groups and reduced movement throughout the EPS. The localisation of PQS at the boundary between infected and non-infected sub-populations, within bacteriophage-infected *P. aeruginosa* biofilms, behaves as a warning signal allowing non-infected bacteria in the immediate neighbourhood to avoid danger^57^. The ability of the PQS molecule to localise and *tether* to the EPS, when the C_4_-HSL molecule does not, suggests that this molecule, perhaps, could be selectively retained. Local PQS retention and local signal accumulation by the EPS has survival advantages and, as these simulations outline, the two oxygen bearing functional groups are of paramount importance to facilitate this.

The EPS-C_4_-HSL and EPS-PQS configurations, which correspond to the minima in the configurational and electrostatic energies are shown, for reference, in Fig. S5. The C_4_-HSL molecule is well separated from the EPS in this configuration, with the distance from its hydrocarbon tail end to the nearest EPS acetyl and EPS-bound Ca^2+^ ion being ~ 16 and ~ 19 Å respectively. However, this configuration, although occupying a minima in the configurational and electrostatic energies and being well separated from the EPS, is not the most thermodynamically stable EPS-C_4_-HSL structure isolated from the simulations. Whilst the C_4_-HSL molecule can readily partition into the solvent phase, it is more thermodynamically stable for the molecule to remain within the vicinity of EPS, albeit not directly interacting with the EPS through ionic or hydrogen bonds. During the C_4_-HSL molecule’s migration over the EPS, it does not interfere significantly with the EPS ionic scaffold, with the average Ca^2+^ coordination numbers, only accounting for EPS oxygen donors, held in the range of 4 to 4.5 throughout the full time-scale of the trajectory. Hence, binding of this molecule is not influenced by the cationic charge distribution in the EPS. Conceivably, therefore, the C_4_-HSL molecule exploits solely Van der Waals (VdW) interactions with the EPS, which are not able to render the molecule immobile at physiological temperature. This, in turn, offers a thermodynamic rationale for the apparent free movement of this molecule. The EPS-PQS adduct corresponding with the minima in the electrostatic and configurational energies (Fig. S5a), encompasses the same ionic and hydrogen bonding molecular binding modes to the EPS as is observed throughout. Unlike the analogous C_4_-HSL structure, this adduct also corresponds to the most thermodynamically stable EPS-PQS system, further accentuating this molecule’s significantly larger propensity to bind to the EPS. It is important to note also, in this configuration, that the PQS $\pi$ system does not align in a fashion to ensure the Ca^2+^ is positioned above its respective plane. As such, the inference is that a quadrupole $\pi$-cation interaction is not a molecular interaction contributing significantly to the overall stability of the EPS-PQS complex. Indeed, previous DFT estimations of $\pi$-cation interactions, across an array of different aromatic systems, have shown that N-heterocyclic aromatics and the presence of electron withdrawing groups, of which both are present in PQS, lower $\pi$-cation binding affinities^58^.

Quorum quenching (QQ) has emerged in recent years as a strategy to limit and/or prevent biofilm proliferation through reducing the concentration of QSAI molecules in the biofilm, through mechanisms such as enzymatic degradation^59^. QQ fails to have any impact on QSAI molecules encompassed within an aqueous phase where the QSAI molecule’s motion is driven by convection^53^. Given the immobility of PQS relative to C_4_-HSL, which can partition into the solvent medium/void spaces, this work proposes PQS as a viable QQ target. In addition, the observed inability of PQS to propagate throughout the exopolysaccharide matrix underscores the requirement for this molecule to be packaged in outer-membrane vesicles (OMVs) if it is to maximise its effectiveness as a (long-range) cell-to-cell signal. Although, not all PQS molecules will be encompassed within OMVs, as it must mediate its own OMV packaging^51,60^ and, understandably, must exist as the free molecule to execute its virulence functions—the inclusion of PQS within OMVs is not associated with biological activity^60^.

Finally, the EPS-PQS adducts are generally, considering the full time-scale of the QSAI trajectories, more thermodynamically stable compared to the EPS-C_4_-HSL systems; the PQS has a higher EPS affinity. Although, in their most stable configurations respectively, the thermodynamic stabilities are comparable. Intriguingly, in their most stable configurations, it is as stable for the C_4_-HSL *to not interact* with the EPS as it is for the PQS molecule *to interact* with the EPS.

## Discussion

This work probed the origin of molecular interactions between quorum sensing autoinducer (QSAI) molecules and the mucoid *P. aeruginosa* exopolysaccharide (EPS) matrix, with the aim of rationalising which molecular interactions govern molecular motion throughout the EPS matrix. To achieve this, a combined molecular dynamics (MD) and Density-Functional Theory (DFT) approach has been employed to identify, and calculate the thermodynamic stability of, EPS-QSAI binding configurations that occur at physiological temperature in the presence of water.

Initially, DFT modelling assisted in the development of a large 4-chain EPS molecular model. These calculations identified that at least two Ca^2+^ ions are needed for the aggregation of four EPS chains and that acetylated copolymeric β-d-mannuronate-α-l-guluronate 4-chain structures are able to facilitate tight complexation about Ca^2+^ ions when the acetyl groups are oppositely displaced. Furthermore, this structure can be distinguished from the less stable acetylated poly-β-d-mannuronate 4-chain analogue through the absence of acetyl contributions to the Ca^2+^ ion chelation geometry. Stable Ca^2+^ cross-linked 4-chain EPS systems show regained egg-box characteristics which were lost at a 2-chain level. The DFT molecular model was transformed, using finite temperature explicit solvent MD, to a molecular model more representative of that which is observed at physiological temperature and in the presence of water. This physiological structure possesses a discontinuous, V-shaped, dendritic morphology arising from a severely reduced intracomplex space. This, in turn, is due to a reduction in the diversity of the Ca^2+^ chelation geometry as a result of carboxylate groups dominating the Ca^2+^ coordination environment over all other oxygen functionality.

A physiologically representative molecular model, combined with the MD-DFT theoretical approach, for the first time has provided atomic-scale chemical insight into the required functional groups for EPS adsorption. The C_4_-HSL molecule interacts with the EPS solely through VdW’s interactions, can partition readily into bulk solvent and void spaces and, is unaffected by the cationic charge distribution. It is most thermodynamically stable for the C_4_-HSL molecule to exist within the vicinity of the EPS and not directly interact which, in turn, offers a thermodynamic rationale for the apparent unperturbed motion of this molecule throughout the biofilm matrix. In contrast, the PQS molecule has the ability to form thermodynamically stable ionic complexes with EPS-bound Ca^2+^ as well as establishing a hydrogen bond directly to a single EPS chain. The PQS hydroxyl group is focal for mediating binding to the EPS and the PQS molecule is rendered immobile through EPS binding. As such, these simulations support the observation that outer-membrane vesicles are required to maximise the effectiveness of PQS as a (long range) cell-to-cell signal. Indeed, OMVs have been implicated in the transportation of PQS, but not the transportation of C_4_-HSL^60^. The MD simulations in this work answer the question, with regards to intermolecular interactions at the atomic-scale, as to why this is the case. With significantly reduced EPS mobility, the PQS molecule is identified as a potential target for quorum quenching (QQ). In fact, enzymatic PQS deactivation, for example, through exogenous supplementation 2,4-dioxygenase, has proved to be a viable strategy for eliminating PQS and PQS related virulence from *P. aeruginosa* biofilms^61,62^.

Finally, the model created and deployed in this work represents the major mucoid *P. aeruginosa* CF lung biofilm matrix component with the correct ionic composition. As such, the molecular interactions between the QSAI’s and the EPS, which occur at physiological equilibrium, captured in these simulations, would occur at greater length scales also. Therefore, these models and simulations provide critical molecular insight into QSAI motion that is equally as applicable when considering QSAI distribution in large complex living biofilms.

## Materials and methods

### Density functional theory (DFT)

Density functional theory (DFT) is a quantum mechanical (computational) approach to accurately predict the energy of a molecular system. Specifically, DFT is grounded on the following principle: that the total energy of a molecular system is a unique functional of the electron density^63^. The true density-functional, however, is unknown and various approximations to the true functional exist, each one being parameterised and made suitable for a particular chemical system. As such, it is important to ensure that the functional of choice has a proven track record in accurately predicting the energy for the system of interest.

The procedure for a DFT calculation begins with the choice of an appropriate density functional and definition of the electron density. The electron density can be written in terms of the wavefunctions which, in turn, are expanded in a basis of plane waves—defined by use of periodic boundary conditions and Bloch’s Theorem^64^. Pseudopotentials are employed to replace the highly oscillatory, and strongly localised, core electron wavefunctions with an electron–ion potential, permitting the use of smaller, more computationally tractable, plane wave basis expansions when describing the electron density^64,65^.

It is important to note that DFT calculations employing semi-local or conventional hybrid density functionals, PBE and B3LYP for example—the popular choice for biomolecular systems—fail to model dispersion interactions. Dispersion interactions, more appropriately, can be defined as the attractive part of the Van der Walls (VdW) interaction potential between atoms and molecules that are not directly bonded. Specifically, these density functionals cannot provide the desired dependence of the dispersion interaction energy on the interatomic distance^66^ and, consequently, energetic predictions on large molecular systems, made using these functionals, are less accurate. To rectify this issue, an empirical potential of the form ${C}_{6}{R}^{-6}$ is added to DFT energy, with *R* being the interatomic distances and ${C}_{6}$ being the dispersion coefficients^67^.

### Computational details

All Density Functional Theory (DFT) calculations were performed using the plane-wave Density Functional Theory (DFT) code, CASTEP^64^. A convergence tested cut-off energy of 900 eV was employed, as well as a Monkhorst–Pack *k*-point grid of 1 × 1 × 1 to sample the Brillouin zone^68^. On-the-fly ultrasoft pseudopotentials were used^69^ alongside the PBE exchange–correlation functional^70^. Intra- and intermolecular dispersive forces were accounted for by applying the semi-empirical dispersion correction of Tkatchenko and Scheffler^71^.

All molecular dynamics trajectories were computed using DL_POLY_4^72^. The conversion of all molecular models into DL_POLY input files was performed using DL_FIELD^73^. Trajectories were computed with the OPLS2005 forcefield^74,75^ in the canonical (NVT) ensemble, where the RATTLE algorithm^76^ was used to constrain covalent bonds to hydrogen, meaning the integration time-step could be increased to 2 ps. The temperature was held at 310 K (body temperature) using Langevin thermostatting^77^. Electrostatics were treated using the Smooth-Particle-Mesh-Ewald method^78^ and the distance cut-offs for electrostatic and Leonard–Jones interactions were set to 1.2 nm.

### Defining an initial starting structure

An upregulation of the alginate biosynthetic gene cluster (*algD*) upon colonisation of the CF lung^79^ offers a mucoid *P. aeruginosa* CF lung biofilm matrix that is predominantly composed of bacterial alginate—an anionic, calcium cross-linked, acetylated polymer of mannuronate (M) and guluronate (G) structures possessing no contiguous G residues^7–10^. Molecular models of thermodynamically stable Ca^2+^ cross-linked acetylated copolymeric $\beta$-d-mannuronate-$\alpha$-l-guluronate (2-PolyMG) and Ca^2+^ cross-linked acetylated poly-$\beta$-d-mannuronate (2-PolyM) 2-chain complexes have previously been developed in the group and validated as being structurally representative of the mucoid *P. aeruginosa* EPS observed in vivo^12^. These models can be seen in Fig. S1 and were a starting point for the development of a larger-scale 4-chain system.

Two 2-PolyMG and two 2-PolyM systems were stacked on top of one another to create two 4-chain systems, a 4-polyMG and a 4-PolyM system. Stacking the 2-chain complexes on top of on another ensured that a fibrillar morphology was maintained as is observed in X-ray diffraction and SAXS measurements of calcium-alginate gels^80,81^. For each 4-chain system, the 2-chain complexes were stacked either parallel or antiparallel along the chain axis with the acetyl groups oriented either parallel or antiparallel. For the 4-PolyM system, which has alternating acetyl orientations, this gave four possible stacking arrangements. In the 4-PolyMG system, which has all of its acetyl groups facing the same orientation, two additional stacking arrangements arose corresponding to the antiparallel acetyl groups both facing towards or away from the neighbouring stack. The stacking arrangements for the 4-PolyMG and 4-PolyM systems are shown in Figs. S2 and S3 respectively.

Each stacking arrangement was subject to a geometry optimisation in a simulation cell measuring 42 Å $\times$ 29 Å $\times$ 45 Å. Due to the large number of different stacking arrangements, an initial screen was performed to identify the most stable stacking arrangement for the 4-PolyMG and 4-PolyM systems. During these screening optimisations, the SCF tolerance was set to $1\times {10}^{-5}$ eV Atom^−1^ and the energy, force and displacement tolerances for the geometry optimisations were set to $5 \times {10}^{-5} \text{eV Atom}^{-1}$, $0.1 \text{eV}$ Å^−1^ and $5 \times {10}^{-3}$ Å respectively. The thermodynamic stability of each 4-chain stacking arrangement was measured by means of evaluating a formation energy (Eq. (1)).

1 $${E}_{f}={E}_{\left\{4-chain\right\}}-{2E}_{\left\{2-chain\right\}}.$$

${E}_{\left\{4-chain\right\}}$ is the energy of the optimised 4-PolyMG or 4-polyM stacking arrangement and ${E}_{\left\{2-chain\right\}}$ is the energy of the initial 2-PolyMG or 2-PolyM system. The formation energies for all stacking arrangements in the 4-PolyMG and 4-PolyM systems are shown in Tables S1 and S2 respectively. While all stacking arrangements were thermodynamically favourable (Eq. (1) returning a negative formation energy), the initial screen highlighted that the most stable arrangement within the 4-PolyMG system involved stacking the chains parallel with the two acetyl groups oriented antiparallel and facing away from adjoining stack. In the 4-PolyM system, the most stable arrangement involved stacking the chains antiparallel with the acetyl groups oriented parallel.

The most stable 4-PolyMG and 4-PolyM stacking arrangements, from here on labelled 4-PolyMG* and 4-PolyM* respectively, were further optimised at finer tolerances to obtain better converged ground-state geometries. For these optimisations the SCF tolerance was set to $2 \times {10}^{-6}$ eV Atom^−1^ and the energy, force and displacement tolerances for the optimisation were set to $2 \times {10}^{-5} \text{eV Atom}^{-1}$, $0.05 \text{eV}$ Å^−1^ and $2 \times {10}^{-3}$ Å respectively. The re-evaluated formation energies (according to Eq. (1)) are − 6.90 eV for the 4-PolyMG* structure and − 6.38 eV for the 4-PolyM* structure, meaning the association of two 2-PolyMG systems is slightly more exothermic compared to the association of two 2-PolyM systems. The 4-PolyMG* structure, therefore, corresponds to the most thermodynamically stable arrangement of four mucoid *P. aeruginosa* EPS chains complexed about Ca^2+^ ions. This structure is shown in Fig. 2 (and for reference the 4-PolyM* structure is shown in Fig. S4).

### From a DFT structure to a physiological structure

The above DFT optimisations were performed at 0 K in vacuo. Therefore, although the above optimisations allowed for the quantification and identification of the most thermodynamically stable arrangement of four mucoid *P. aeruginosa* EPS chains complexed about Ca^2+^ ions, they fail to reflect possible conformational changes that could occur at physiological temperature and in the presence of water.

The 4-PolyMG* structure was, therefore, used as an initial (starting) structure for a subsequent MD simulation to obtain a structure more representative of that observed at physiological temperature. This MD simulation was performed over 2 ns under periodic boundary conditions based on a simulation cell measuring 60 Å $\times$ 60 Å $\times$ 60 Å encompassing the 4-PolyMG* structure and SPC (Simple Point Charge) water. The thermally equilibrated 4-PolyMG* structure, from here on, is labelled as 4-PolyMG_MD_ and can be seen in Fig. 3. This structure corresponds to a complete molecular model of a hydrated mucoid *P. aeruginosa* exopolysaccharide (EPS) matrix which exists at physiological temperature.

### EPS-QSAI simulations

MD simulations were performed to sample modes of interaction between the 4-PolyMG_MD_ structure and two QSAI molecules, C_4_-HSL and PQS (Fig. 1). To get structures of these molecules that are representative of the conformations observed at physiological temperature, these two molecules were equilibrated over 1 ns under periodic boundary conditions in a simulation cell measuring 40 Å $\times$ 40 Å $\times$ 40 Å, solvated with SPC water.

After obtaining thermally equilibrated conformations for the two QSAI molecules, they were individually combined with 4-PolyMG_MD_ structure, positioned (docked) 6 Å away from the base of a V-shaped cleft that opened up during its thermal equilibration at 310 K (see Fig. 3). MD trajectories were computed over 10 ns under periodic boundary conditions in a simulation cell measuring 60 Å $\times$ 60 Å $\times$ 60 Å solvated with SPC water. This trajectory length was suitable to allow the molecule to sample preferential interaction modes/binding sites. Structures (EPS-molecule adducts) were isolated from the trajectories every 1 ns, as well as at the time-step corresponding to a minima in the configurational and electrostatic energies, for subsequent DFT thermodynamic stability calculations.

### Thermodynamic stability of the EPS-QSAI adducts

If the isolated EPS-QSAI structures were separated by $\le$ 6 Å, their thermodynamic stabilities were evaluated by means of evaluating a formation energy (Eq. (2)).

2 $${E}_{f}={E}_{\left\{matrix-molecule\, adduct\right\}}-\left({E}_{\left\{4-PolyM{G}_{\left\{MD\right\}}\right\}}+{E}_{\left\{molecule\right\}}\right).$$

${E}_{\left\{matrix-molecule \,adduct\right\}}$ is the energy of an isolated EPS-molecule system, ${E}_{\left\{4-PolyM{G}_{\left\{MD\right\}}\right\}}$ is the energy of the thermally equilibrated 4-PolyMG_MD_ system and ${E}_{\left\{molecule\right\}}$ is the energy of a thermally equilibrated molecule, either C_4_-HSL or PQS. The energy of each system (each term in Eq. (2)) was evaluated using 0 K in vacuo DFT single-point energy calculations where the SCF tolerance was set to $2 \times {10}^{-6}$ eV Atom^−1^. Finally, Mulliken bond populations^82^ were calculated to classify the nature of bonding between the EPS and the molecule in each of the final EPS-molecule adducts.

## Supplementary Information

Supplementary Information.

## Data Availability

All data generated or analysed during this study are included in this published article (and its Supplementary Information files).

## References

[1] Parkins MD, Somayaji R, Waters VJ (2018). Epidemiology, biology, and impact of clonal *Pseudomonas aeruginosa* infections in cystic fibrosis. Clin. Microbiol. Rev..

[2] Lyczak JB, Cannon CL, Pier GB (2000). Establishment of *Pseudomonas aeruginosa* infection: Lessons from a versatile opportunist. Microbes. Infect..

[3] Hall-Stoodley L, Stoodley P (2009). Evolving concepts in biofilm infections. Cell. Microbiol..

[4] Costerton JW, Lewandowski Z, Caldwell DE, Korber DR, Lappin-Scott HM (1995). Microbial biofilms. Annu. Rev. Microbiol..

[5] Karygianni L, Ren Z, Koo H, Thurnheer T (2020). Biofilm matrixome: Extracellular components in structured microbial communities. Trends Microbiol..

[6] Bjarnsholt T (2009). *Pseudomonas aeruginosa* biofilms in the respiratory tract of cystic fibrosis patients. Pediatr. Pulmonol..

[7] SkjÅk-Bræk G, Paoletti S, Gianferrara T (1989). Selective acetylation of mannuronic acid residues in calcium alginate gels. Carbohydr. Res..

[8] SkjÅk-Bræk G, Grasdalen H, Larsen B (1986). Monomer sequence and acetylation pattern in some bacterial alginates. Carbohydr. Res..

[9] Linker A, Jones RS (1966). A new polysaccharide resembling alginic acid isolated from *Pseudomonads*. J. Biol. Chem..

[10] Evans LR, Linker A (1973). Production and characterization of the slime polysaccharide of *Pseudomonas aeruginosa*. J. Bacteriol..

[11] Smith DJ, Anderson GJ, Bell SC, Reid DW (2014). Elevated metal concentrations in the CF airway correlate with cellular injury and disease severity. J. Cyst. Fibros.

[12] Hills OJ, Smith J, Scott AJ, Devine DA, Chappell HF (2021). Cation complexation by mucoid *Pseudomonas aeruginosa* extracellular polysaccharide. PLoS ONE.

[13] Smith RS, Iglewski BH (2003). *P. aeruginosa* quorum-sensing systems and virulence. Curr. Opin. Microbiol..

[14] Davies DG (1998). The involvement of cell-to-cell signals in the development of a bacterial biofilm. Science.

[15] De Kievit TR, Iglewski BH (2000). Bacterial quorum sensing in pathogenic relationships. Infect. Immun..

[16] Darch SE (2018). Spatial determinants of quorum signaling in a *Pseudomonas aeruginosa infection* model. PNAS.

[17] Singh PK (2000). Quorum-sensing signals indicate that cystic fibrosis lungs are infected with bacterial biofilms. Nature.

[18] Lawrence JR, Swerhone GDW, Kuhlicke U, Neu TR (2007). In situ evidence for microdomains in the polymer matrix of bacterial microcolonies. Can. J. Microbiol..

[19] Tan CH (2014). The role of quorum sensing signalling in EPS production and the assembly of a sludge community into aerobic granules. ISME J..

[20] da Silva DP, Schofield MC, Parsek MR, Tseng BS (2017). An update on the sociomicrobiology of quorum sensing in gram-negative biofilm development. Pathogens.

[21] Parsek MR, Greenberg EP (2005). Sociomicrobiology: The connections between quorum sensing and biofilms. Trends Microbiol..

[22] Whiteley M, Greenberg EP (2001). Promoter specificity elements in *Pseudomonas aeruginosa* quorum-sensing-controlled genes. J. Bacteriol..

[23] Erickson DL (2002). *Pseudomonas aeruginosa* quorum-sensing systems may control virulence factor expression in the lungs of patients with cystic fibrosis. Infect. Immun..

[24] Favre-Bonté S, Köhler T, Van Delden C (2003). Biofilm formation by *Pseudomonas aeruginosa*: Role of the C4-HSL cell-to-cell signal and inhibition by azithromycin. J. Antimicrob. Chemother..

[25] Seed PC, Passador L, Iglewski BH (1995). Activation of the *Pseudomonas aeruginosa* lasI gene by LasR and the *Pseudomonas* autoinducer PAI: An autoinduction regulatory hierarchy. J. Bacteriol..

[26] Schuster M, Lostroh CP, Ogi T, Greenberg EP (2003). Identification, timing, and signal specificity of *Pseudomonas aeruginosa* quorum-controlled genes: A transcriptome analysis. J. Bacteriol..

[27] Alayande AB, Aung MM, Kim IS (2018). Correlation between quorum sensing signal molecules and *Pseudomonas aeruginosa’s* biofilm development and virulency. Curr. Microbiol..

[28] Pesci EC (1999). Quinolone signaling in the cell-to-cell communication system of *Pseudomonas aeruginosa*. Proc. Natl. Acad. Sci. U.S.A..

[29] Collier DN (2002). A bacterial cell to cell signal in the lungs of cystic fibrosis patients. FFEMS Microbiol. Lett..

[30] McKnight SL, Iglewski BH, Pesci EC (2000). The *Pseudomonas* quinolone signal regulates rhl quorum sensing in *Pseudomonas aeruginosa*. J. Bacteriol..

[31] D’Argenio DA, Calfee MW, Rainey PB, Pesci EC (2002). Autolysis and autoaggregation in *Pseudomonas aeruginosa* colony morphology mutants. J. Bacteriol..

[32] Popat R (2017). Environmental modification via a quorum sensing molecule influences the social landscape of siderophore production. Proc. R. Soc. B.

[33] Bredenbruch F, Geffers R, Nimtz M, Buer J, Häussler S (2006). The *Pseudomonas aeruginosa* quinolone signal (PQS) has an iron-chelating activity. Environ. Microbiol..

[34] Diggle SP (2007). The *Pseudomonas aeruginosa* 4-quinolone signal molecules HHQ and PQS play multifunctional roles in quorum sensing and iron entrapment. Chem. Biol..

[35] Davey ME, Caiazza NC, O’Toole GA (2003). Rhamnolipid surfactant production affects biofilm architecture in *Pseudomonas aeruginosa* PAO1. J. Bacteriol..

[36] Kim H-S, Lee S-H, Byun Y, Park H-D (2015). 6-Gingerol reduces *Pseudomonas aeruginosa* biofilm formation and virulence via quorum sensing inhibition. Sci. Rep..

[37] Nain Z, Sayed SB, Karim MM, Islam MA, Adhikari UK (2019). Energy-optimized pharmacophore coupled virtual screening in the discovery of quorum sensing inhibitors of LasR protein of *Pseudomonas aeruginosa*. J. Biomol. Struct. Dyn..

[38] Hnamte S (2019). Mosloflavone attenuates the quorum sensing controlled virulence phenotypes and biofilm formation in *Pseudomonas aeruginosa* PAO1: In vitro, in vivo and in silico approach. Microb. Pathog..

[39] Menakbi C, Quignard F, Mineva T (2016). Complexation of trivalent metal cations to mannuronate type alginate models from a density functional study. J. Phys. Chem. B.

[40] Stewart MB, Gray SR, Vasiljevic T, Orbell JD (2014). The role of poly-M and poly-GM sequences in the metal-mediated assembly of alginate gels. Carbohydr. Polym..

[41] Lawrence JR, Korber DR, Hoyle BD, Costerton JW, Caldwell DE (1991). Optical sectioning of microbial biofilms. J. Bacteriol..

[42] Vogt M, Flemming H, Veeman WS (2000). Diffusion in *Pseudomonas aeruginosa* biofilms: A pulsed field gradient NMR study. J. Biotechnol..

[43] Xiang Y, Liu Y, Mi B, Leng Y (2014). Molecular dynamics simulations of polyamide membrane, calcium alginate gel, and their interactions in aqueous solution. Langmuir.

[44] Plazinski W (2011). Molecular basis of calcium binding by polyguluronate chains. Revising the egg-box model. J. Comput. Chem..

[45] Plazinski W, Drach M (2012). The dynamics of the calcium-induced chain–chain association in the polyuronate systems. J. Comput. Chem..

[46] Grant GT, Morris ER, Rees DA, Smith PJC, Thom D (1973). Biological interactions between polysaccharides and divalent cations: The egg-box model. FEBS. Lett..

[47] Hunter RC, Beveridge TJ (2005). High-resolution visualization of *Pseudomonas aeruginosa* PAO1 biofilms by freeze-substitution transmission electron microscopy. J. Bacteriol..

[48] Ritenberg M (2016). Imaging *Pseudomonas aeruginosa* biofilm extracellular polymer scaffolds with amphiphilic carbon dots. ACS Chem. Biol..

[49] Stewart MB, Gray SR, Vasiljevic T, Orbell JD (2014). Exploring the molecular basis for the metal-mediated assembly of alginate gels. Carbohydr. Polym..

[50] Hecht H, Srebnik S (2016). Structural characterization of sodium alginate and calcium alginate. Biomacromol.

[51] Mashburn-Warren L (2008). Interaction of quorum signals with outer membrane lipids: Insights into prokaryotic membrane vesicle formation. Mol. Microbiol..

[52] Flemming H-C (2016). Biofilms: An emergent form of bacterial life. Nat. Rev. Microbiol..

[53] Tan CH (2020). Convection and the extracellular matrix dictate inter- and intra-biofilm quorum sensing communication in environmental systems. Environ. Sci. Technol..

[54] Sankaran J (2019). Single microcolony diffusion analysis in *Pseudomonas aeruginosa* biofilms. NPJ Biofilms Microbiomes.

[55] Baig FN (2015). Multimodal chemical imaging of molecular messengers in emerging *Pseudomonas aeruginosa* bacterial communities. Analyst.

[56] Morales-Soto N (2018). Spatially dependent alkyl quinolone signaling responses to antibiotics in *Pseudomonas aeruginosa* swarms. J. Biol. Chem..

[57] Bru JL (2019). PQS produced by the *Pseudomonas aeruginosa* stress response repels swarms away from bacteriophage and antibiotics. J. Bacteriol..

[58] Mecozzi S, West A, Dougherty D (1996). Cation-π interactions in aromatics of biological and medicinal interest: Electrostatic potential surfaces as a useful qualitative guide. Proc. Natl. Acad. Sci. U.S.A..

[59] Paluch E, Rewak-Soroczyńska J, Jędrusik I, Mazurkiewicz E, Jermakow K (2020). Prevention of biofilm formation by quorum quenching. Appl. Microbiol. Biotechnol..

[60] Mashburn LM, Whiteley M (2005). Membrane vesicles traffic signals and facilitate group activities in a prokaryote. Nature.

[61] Pustelny C (2009). Dioxygenase-mediated quenching of quinolone-dependent quorum sensing in *Pseudomonas aeruginosa*. Chem. Biol..

[62] Arranz San Martín A, Vogel J, Wullich SC, Quax WJ, Fetzner S (2022). Enzyme-mediated quenching of the *pseudomonas* quinolone signal (PQS): A comparison between naturally occurring and engineered PQS-cleaving dioxygenases. Biomolecules.

[63] Hohenberg P, Kohn W (1964). Inhomogeneous electron gas. Phys. Rev..

[64] Clark SJ (2005). First principles methods using CASTEP. Z. Krist..

[65] Segall MD (2002). First-principles simulation: Ideas, illustrations and the CASTEP code. J. Phys. Condens. Matter..

[66] Grimme S (2011). Density functional theory with London dispersion corrections. Wiley Interdiscip. Rev. Comput. Mol. Sci..

[67] Grimme S (2004). Accurate description of van der Waals complexes by density functional theory including empirical corrections. J. Comput. Chem..

[68] Monkhorst HJ, Pack JD (1976). Special points for Brillouin-zone integrations. Phys. Rev. B.

[69] Vanderbilt D (1990). Soft self-consistent pseudopotentials in a generalized eigenvalue formalism. Phys. Rev. B.

[70] Perdew JP, Burke K, Ernzerhof M (1996). Generalized gradient approximation made simple. Phys. Rev. Lett..

[71] Tkatchenko A, Scheffler M (2009). Accurate molecular van der Waals interactions from ground-state electron density and free-atom reference data. Phys. Rev. Lett..

[72] Todorov IT, Smith W, Trachenko K, Dove MT (2006). DL_POLY_3: New dimensions in molecular dynamics simulations via massive parallelism. J. Mater. Chem..

[73] Yong CW (2016). Descriptions and implementations of DL_F notation: A natural chemical expression system of atom types for molecular simulations. J. Chem. Inf. Model..

[74] Jorgensen WL, Maxwell DS, Tirado-Rives J (1996). Development and testing of the OPLS all-atom force field on conformational energetics and properties of organic liquids. J. Am. Chem. Soc..

[75] Banks JL (2005). Integrated modeling program, applied chemical theory (IMPACT). J. Comput. Chem..

[76] Andersen HC (1983). Rattle: A “velocity” version of the shake algorithm for molecular dynamics calculations. J. Comput. Phys..

[77] Adelman SA, Doll JD (1976). Generalized Langevin equation approach for atom/solid-surface scattering: General formulation for classical scattering off harmonic solids. J. Chem. Phys..

[78] Essmann U (1998). A smooth particle mesh Ewald method. J. Chem. Phys..

[79] Chitnis CE, Ohman DE (1993). Genetic analysis of the alginate biosynthetic gene cluster of *Pseudomonas aeruginosa* shows evidence of an operonic structure. Mol. Microbiol..

[80] Agulhon P, Robitzer M, David L, Quignard F (2012). Structural regime identification in ionotropic alginate gels: Influence of the cation nature and alginate structure. Biomacromol.

[81] Sikorski P, Mo F, Skjåk-Bræk G, Stokke BT (2007). Evidence for egg-box-compatible interactions in calcium-alginate gels from fiber X-ray diffraction. Biomacromol.

[82] Mulliken RS (1955). Electronic population analysis on LCAO–MO molecular wave functions. I. J. Chem. Phys..

