# Supplementary material for: Atomic-scale interactions between quorum sensing autoinducer molecules and the mucoid P. aeruginosa exopolysaccharide matrix
Source: Sci Rep. 2022 May 11;12:7724. doi: 10.1038/s41598-022-11499-9 (PMC9095684; doi:10.1038/s41598-022-11499-9)
Supplement: Supplementary file 1 — Supplementary Information. [file 41598_2022_11499_MOESM1_ESM.docx]

**Supplementary Information for**

**Atomic-scale interactions between quorum sensing autoinducer molecules and the mucoid *P. aeruginosa* exopolysaccharide matrix**

Oliver J. Hills^1*^, Chin W. Yong^2,3^ , Andrew J. Scott^4^, Deirdre A. Devine^5^, James Smith^1^ & Helen F. Chappell^1*^

^1^School of Food Science & Nutrition, University of Leeds, Woodhouse Lane, Leeds, LS2 9JT, UK

^2^Scientific Computing Department, Science and Technology Facilities Council, Daresbury Laboratory, Keckwick Lane, Daresbury, Warrington, WA4 4AD, UK

^3^Division of Pharmacy and Optometry, School of Health Sciences, University of Manchester, Oxford Road, Manchester M13 9PL, UK

^4^School of Chemical & Process Engineering, University of Leeds, Woodhouse Lane, Leeds, LS2 9JT, UK

^5^School of Dentistry, University of Leeds, Clarendon Way, LS2 9LU, UK

*Corresponding authors: Oliver J. Hills, Helen F. Chappell

**Email:**  Email: [fsojh@leeds.ac.uk](mailto:fsojh@leeds.ac.uk) (OJH) & [H.F.Chappell@leeds.ac.uk](mailto:H.F.Chappell@leeds.ac.uk) (HFC)

Supplementary text

Shown in Fig S1 are the molecular structures for the 2-polyMG and 2-PolyM systems developed previously within the group (reference 12 in main text).

Shown in Fig S2 and Fig S3 are the different possible stacking arrangements tested when identifying the most thermodynamically stable packing arrangement of four mucoid *P. aeruginosa* exopolysaccharide chains complexed about Ca^2+^ ions.

The formation energies, a measure of thermodynamic stability, for all stacking arrangements for the 4-PolyMG and 4-PolyM systems are shown in Table S1 and Table S2 respectively. The 4-PolyM* structure is shown Fig S4.

Shown in Table S3 are the geometrical features, namely the average oxygen-Ca^2+^ bond lengths and populations for all coordinating oxygen functional groups, in the 4-PolyMG* and 4-PolyM* systems.

Shown in Fig S5 are the EPS-C_4_-HSL and EPS-PQS configurations which correspond to the minima in the configurational and electrostatic energies.

Materials and Methods

**Defining an initial starting structure**

| 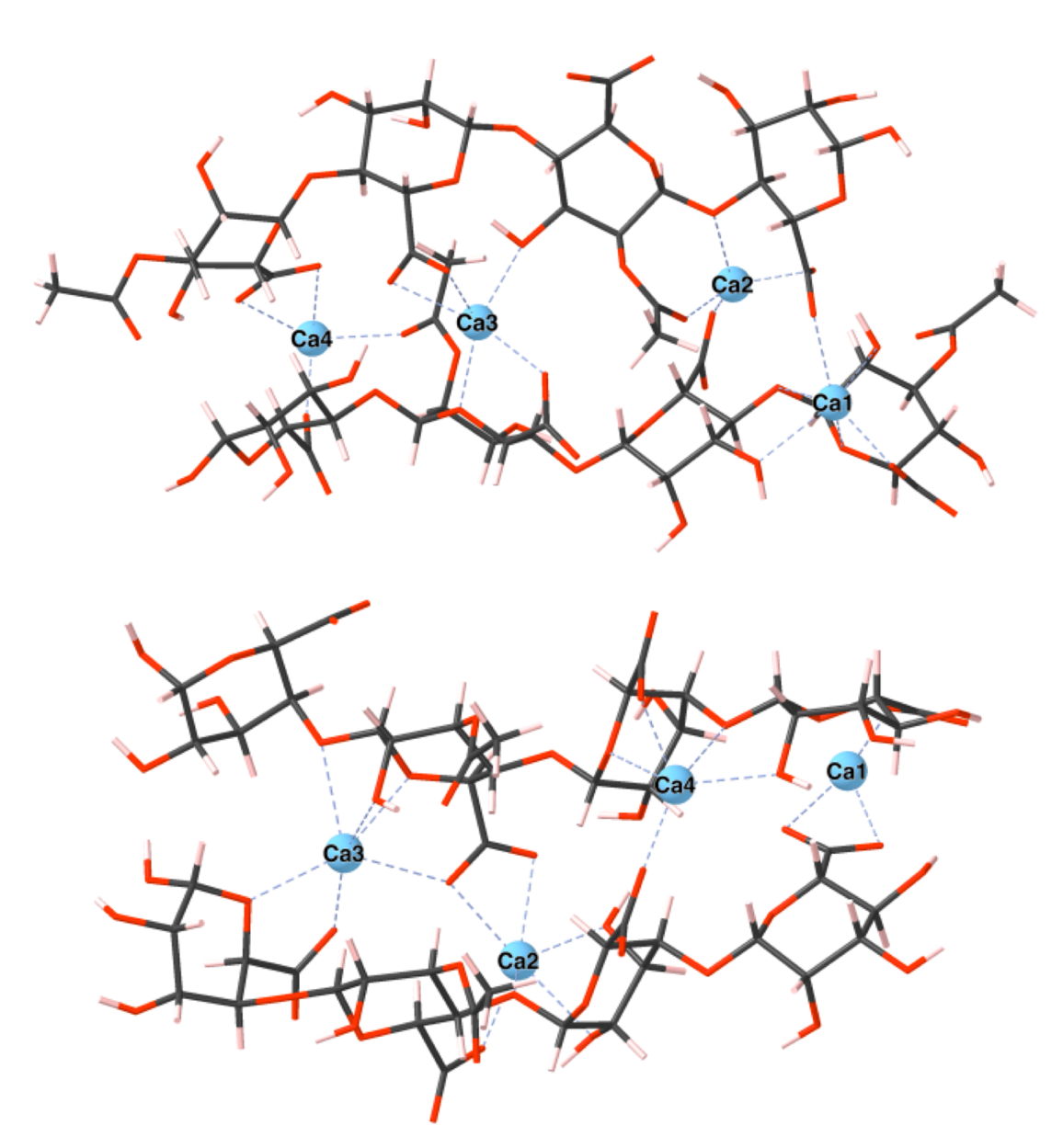 |
| --- |
| **Fig S1.** 2-PolyM (Top) and 2PolyMG (bottom) complexes. Carbon atoms are shown in grey, oxygen in red, calcium in blue and hydrogen in pink. Calcium ions are labelled and calcium-oxygen ionic bonds are shown with dashed blue lines. |


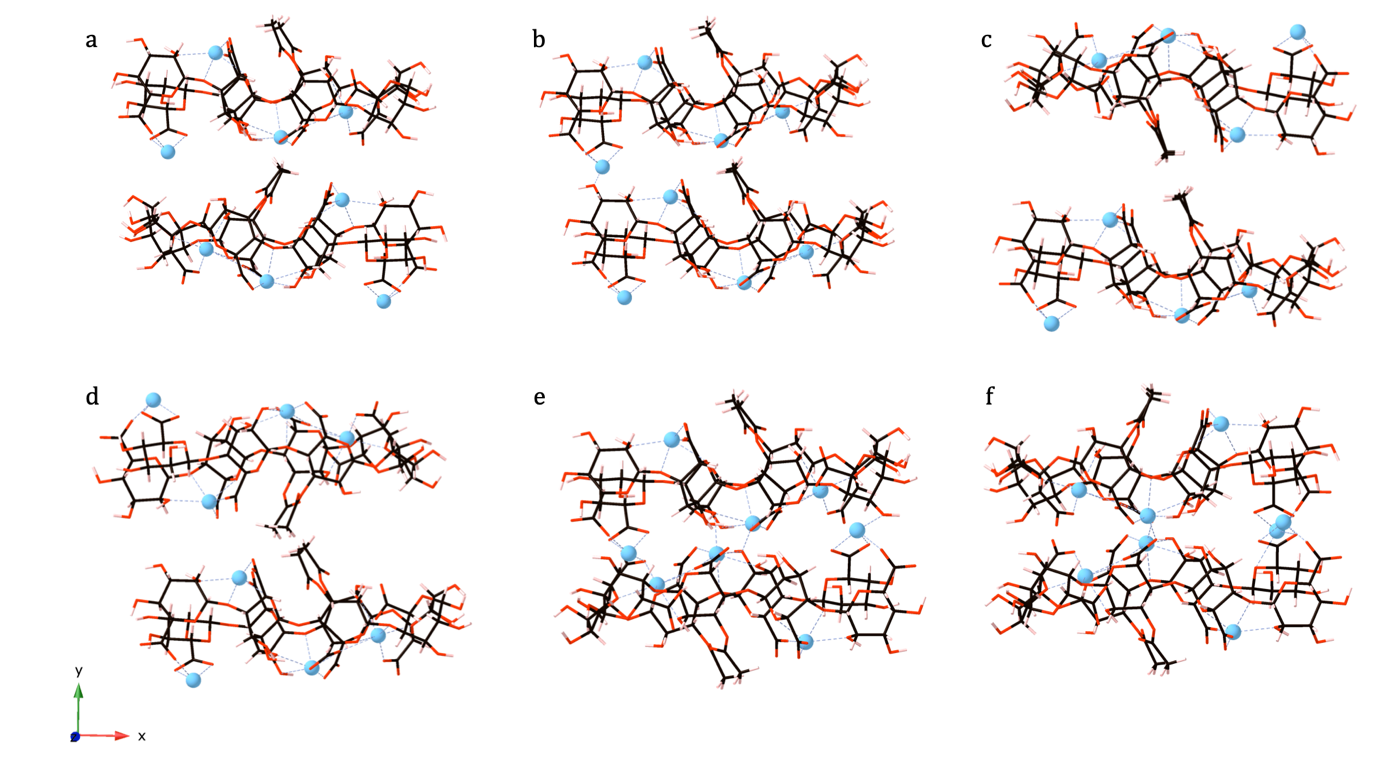


Fig S2. Different stacking arrangements tested when identifying the most thermodynamically stable packing arrangement in the 4-PolyMG system: a) Chains antiparallel, acetyl parallel, b) Chains parallel, acetyl parallel, c) Chains antiparallel, acetyl antiparallel facing towards neighbouring stack, d) Chains parallel, acetyl antiparallel facing towards neighbouring stack, e) Chains antiparallel, acetyl antiparallel facing away from neighbouring stack & f) Chains parallel, acetyl antiparallel facing away from neighbouring stack. Carbon atoms are shown in grey, oxygen in red, calcium in blue and hydrogen in pink. Calcium-oxygen bonds are shown with dashed blue lines.


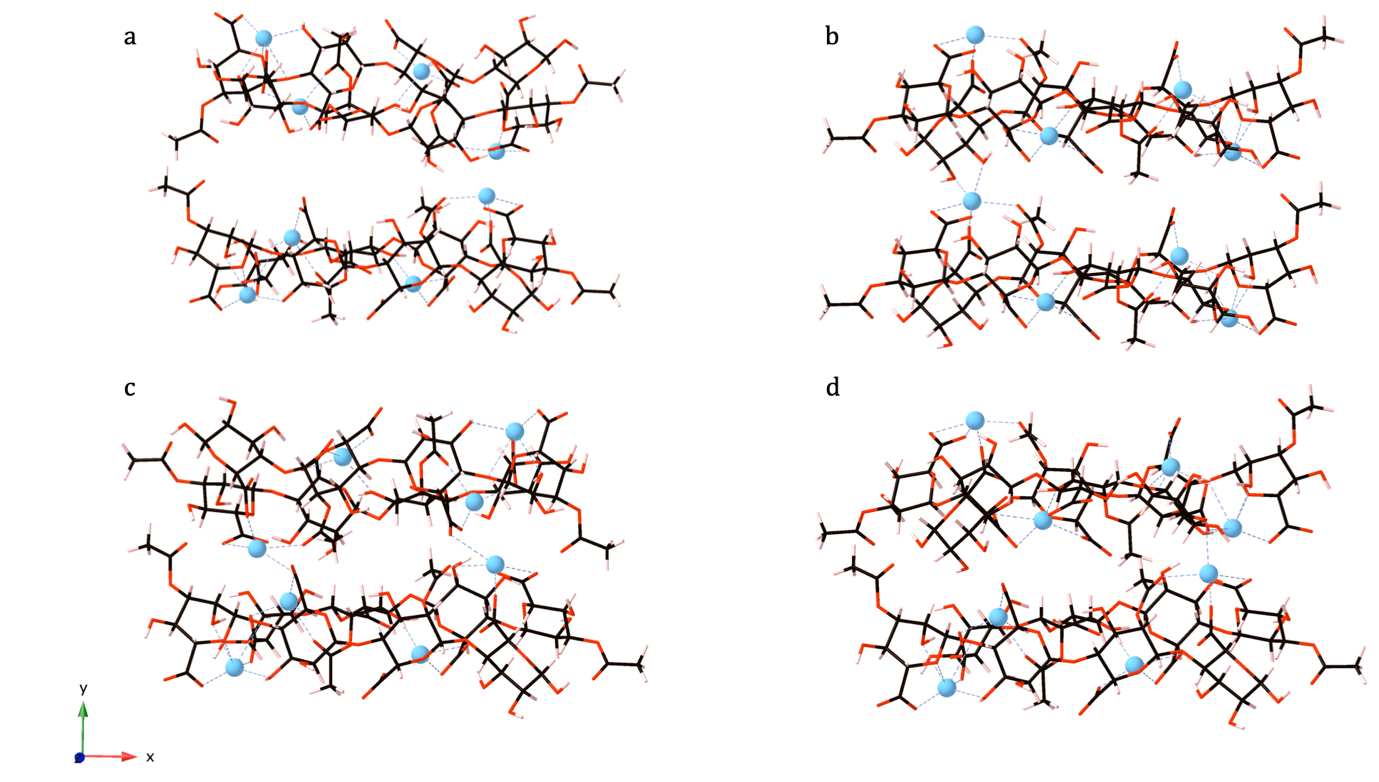


Fig S3. Different stacking arrangements tested when identifying the most thermodynamically stable packing arrangement in the 4-PolyM system: a) Chains parallel, acetyl antiparallel, b) Chains parallel, acetyl parallel, c) Chains antiparallel, acetyl parallel & d) Chains antiparallel, acetyl antiparallel . Carbon atoms are shown in grey, oxygen in red, calcium in blue and hydrogen in pink. Calcium-oxygen bonds are shown with dashed blue lines.

**
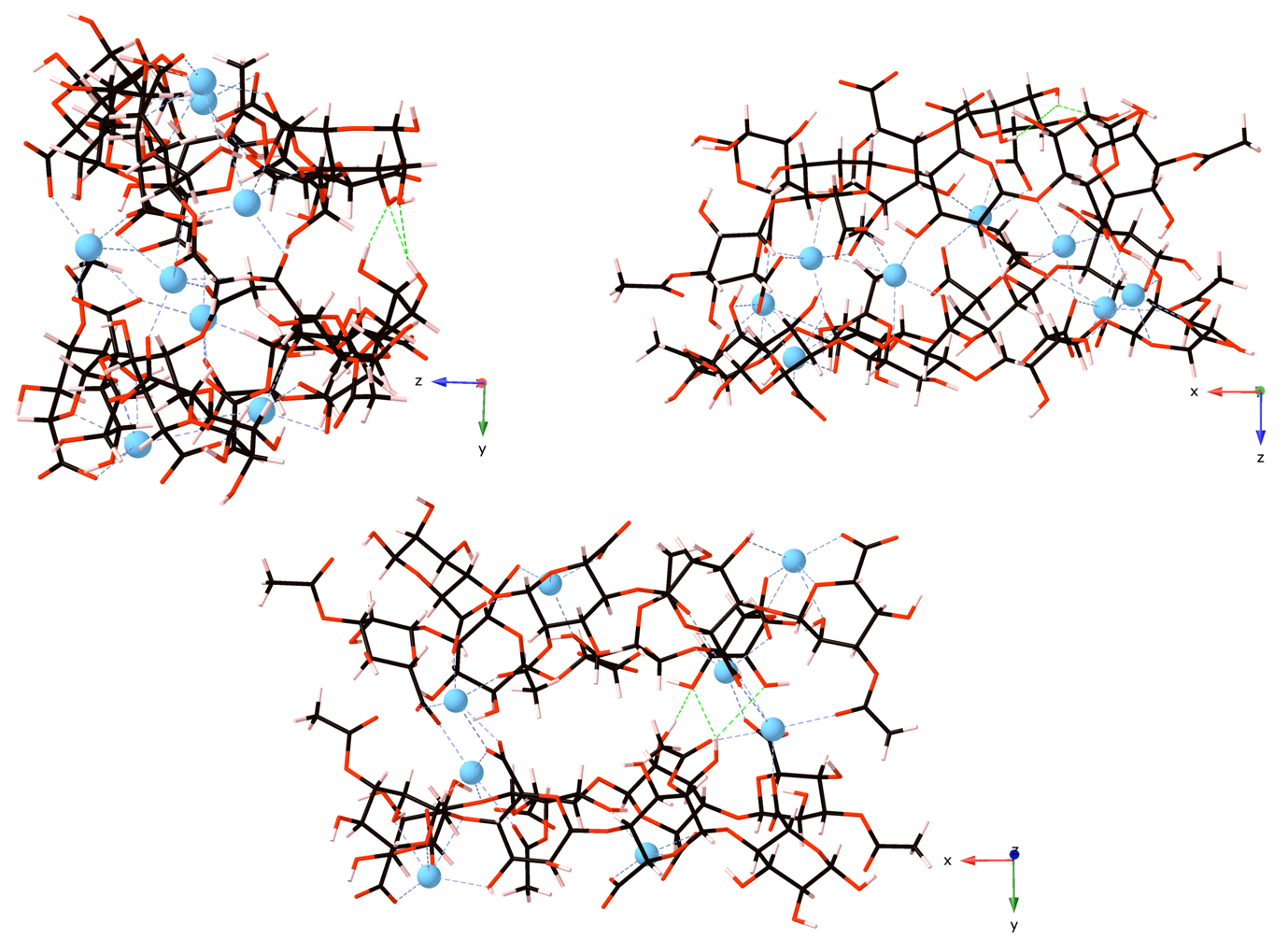
**

**Fig S4**: The 4-PolyM* structure viewed down the x, y and z axes. Carbon atoms are shown in grey, oxygen in red, calcium in blue and hydrogen in pink. Calcium-oxygen ionic bonds are shown with dashed blue lines and hydrogen bonds are shown with dashed green lines.

Results

Exothermic association of four EPS chains about Ca^2+^ ions

Table S1: Formation energies (eV), number of ionic bonds between adjoining stacks and number of hydrogen bonds between adjoining stacks for all stacking arrangements in the 4-PolyMG system.

| **System** | **E_f_ (eV)** | **Number of ionic bonds between adjoining stacks** | **Number of hydrogen bonds between adjoining stacks** |
| --- | --- | --- | --- |
| Chains antiparallel, acetyls parallel | -1.75 | 1 | 0 |
| Chains parallel, acetyls parallel | -1.81 | 2 | 1 |
| Chains antiparallel, both acetyls antiparallel facing towards neighbouring stack | -0.17 | 0 | 0 |
| Chains parallel, both acetyls antiparallel facing towards neighbouring stack | -0.52 | 0 | 0 |
| Chains antiparallel, both acetyls antiparallel facing away from neighbouring stack | -5.11 | 5 | 1 |
| Chains parallel, both acetyls antiparallel facing away from neighbouring stack | -6.88 | 8 | 3 |

**Table S2**: Formation energies (eV), number of ionic bonds between adjoining stacks and number of hydrogen bonds between adjoining stacks for all stacking arrangements in the 4-PolyM system.

| **System** | **E_f_ (eV)** | **Number of ionic bonds between adjoining stacks** | **Number of hydrogen bonds between adjoining stacks** |
| --- | --- | --- | --- |
| Chains parallel, acetyl antiparallel | -1.44 | 1 | 1 |
| Chains parallel, acetyl parallel | -2.84 | 3 | 0 |
| Chains antiparallel, acetyl parallel | -6.20 | 7 | 3 |
| Chains antiparallel, acetyl antiparallel | -2.83 | 3 | 1 |

**Table S3**: Average oxygen-Ca^2+^ bond lengths and populations for the 4-PolyMG* and 4-PolyM* systems.

| 4-PolyMG* | | |
| --- | --- | --- |
| O-Ca^2+^ contacts | Average length (Å) | Average population (\|e\|) |
| O-Ca^2+^ | 2.41 | 0.10 |
| COO-Ca^2+^ | 2.33 | 0.11 |
| OH-Ca^2+^ | 2.50 | 0.088 |
| Ring O-Ca^2+^ | 2.52 | 0.094 |
| Glycosidic O-Ca^2+^ | 2.49 | 0.11 |
| 4-PolyM* | | |
| O-Ca^2+^ contacts | Average length (Å) | Average population (\|e\|) |
| O-Ca^2+^ | 2.39 | 0.11 |
| COO-Ca^2+^ | 2.31 | 0.13 |
| OH-Ca^2+^ | 2.50 | 0.088 |
| Ring O-Ca^2+^ | 2.48 | 0.088 |
| Glycosidic O-Ca^2+^ | 2.43 | 0.088 |
| Acetyl O-Ca^2+^ | 2.55 | 0.092 |

QSAI simulations


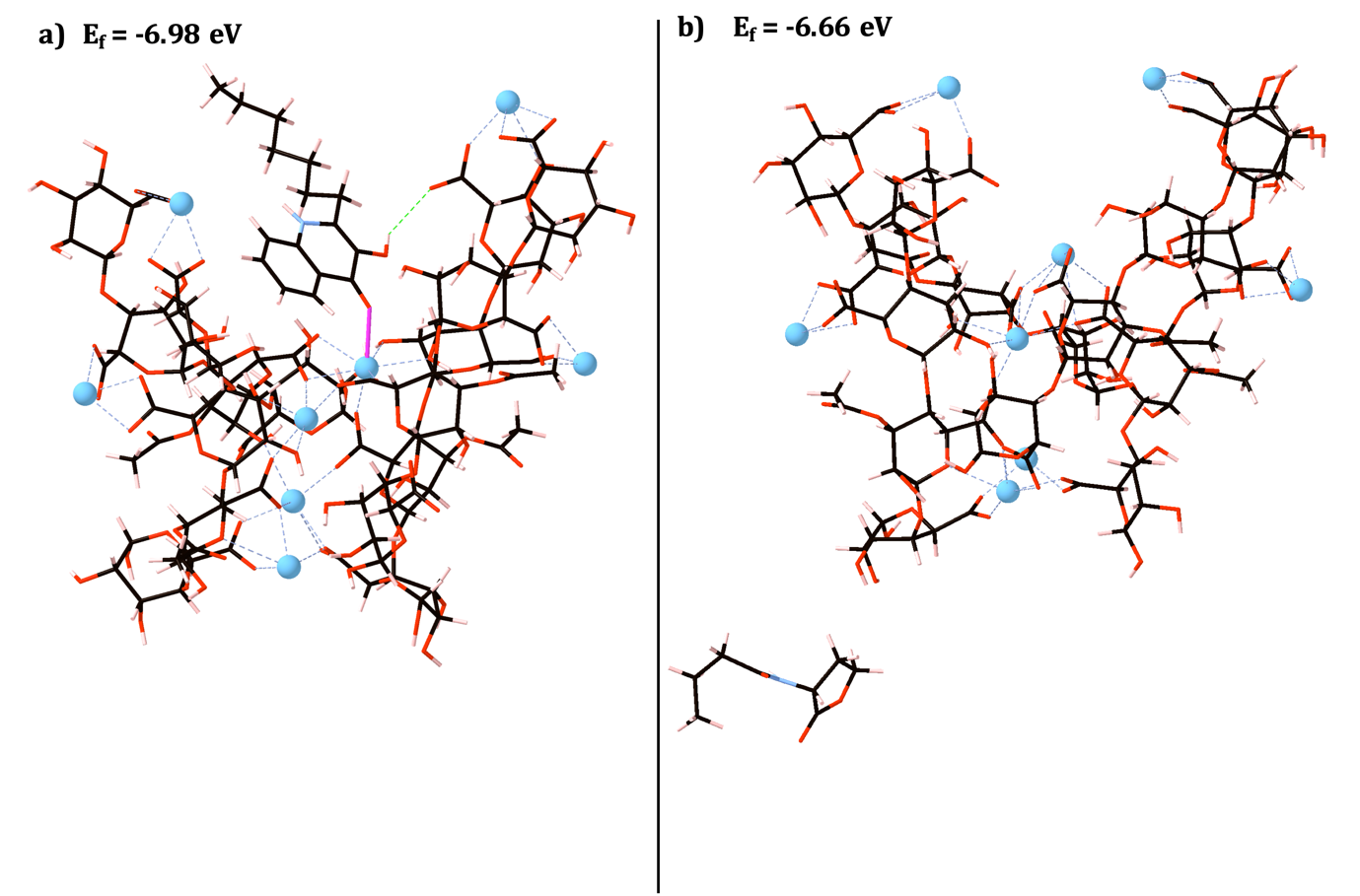


**Fig S5**: The EPS-PQS adduct (a) and EPS-C_4_-HSL adduct (b) which correspond to the minima in the electrostatic and configurational energies. The associated formation energies (evaluated according to Equation 2) are also given. Carbon atoms are shown in grey, oxygen in red, calcium in blue and hydrogen in pink. Calcium-oxygen ionic bonds are shown with dashed blue lines and hydrogen bonds are shown with dashed green lines. Ionic bonds between the PQS and 4-PolyMG­_MD_ system are shown with bold pink lines.
